# Characterization of circular RNAs in mammary tissue from Holstein cows at perinatal period and dry period

**DOI:** 10.3389/fgene.2026.1835098

**Published:** 2026-05-26

**Authors:** Yan Liang, Shuang Gu, Zhaozheng Zhang, Yanru Wang, Mingxun Li, Niel A. Karrow, Jinling Hua, Yongjiang Mao

**Affiliations:** 1 College of Animal Science, Anhui Science and Technology University, Fengyang, China; 2 College of Animal Science and Technology, Yangzhou University, Yangzhou, China; 3 Center for Genetic Improvement of Livestock, Department of Animal Biosciences, University of Guelph, Guelph, ON, Canada

**Keywords:** circular RNAs, dry period, Holstein cows, mammary tissue, perinatal period, RNA sequencing

## Abstract

**Introduction:**

Perinatal and dry periods are important physiological stages for cows to recover after calving and improve lactation performance. Exploring the expression characteristics of circRNAs as a molecular hotspot during the perinatal and dry periods of dairy cows is of great significance.

**Methods:**

This study identified and compared circular RNAs (circRNAs) in the mammary tissue of three cows between the perinatal and dry periods. After analysis, we identified 10,388 circRNAs, ranging from 48 bp to 99,406 bp.

**Results:**

Chromosome 1 had the most circRNAs, containing 597 circRNAs. Furthermore, 91.97% of the circRNAs belonged to sense-overlapping circRNA. CircRNAs contain different number of exons, ranging from 1 to 47 and most of the cirsRNAs harbored 1 to four exons. Compared with dry period, 132 circRNAs with significantly different expressions were identified in the perinatal period, 99 of which were upregulated and 33 downregulated. Enrichment analysis revealed the enrichment of circRNAs in the proliferation and differentiation of cells, such as regulation of chondrocyte differentiation and integral component of plasma membrane, phosphatidylinositol 3-kinase binding. The significantly enriched pathways further indicate that circRNAs play important roles in immunity and infection, such as cell adhesion molecules (CAMs), herpes simplex infection, and Kaposi’s sarcoma-associated herpesvirus infection.

**Discussion:**

This study revealed the expression profile and characteristics of circRNAs in the perinatal and dry period of Holstein cows, thus providing rich information for studying circRNAs functions and mechanisms underlying perinatal and dry period diseases, suggesting a new avenue to investigate the regulatory mechanisms of dairy cow genetic breeding.

## 1 Introduction

The perinatal period of dairy cows (includes the 3 weeks before and after parturition) is the most important in their lives, closely related to their lactation and reproductive performance (Drackley, 1999). During this period, a series of significant changes have taken place in their hormone levels, rumen function, nutritional metabolism, and immunity (Mulligan and Doherty, 2008; LeBlanc et al., 2006). This prompted considerable research efforts in this area aimed at investigating physiological changes in cows during the perinatal period. These studies have shown that even in the absence of microbial infection and/or other identified signs of pathology, perinatal cows exhibit a pronounced inflammatory response, which increases metabolic stress and thus damages the host’s immune defenses (Trevisi et al., 2012). The transcriptomic analysis revealed that the cell cycle, DNA damage, and chromosomal conformation of perinatal neutrophils in dairy cows are strongly associated with the incidence of mastitis during lactation (Jiang et al., 2022). The dry period (traditionally about 6–8 weeks) has important impacts on the recovery of udders, limbs and feet, and rumen recovery, and can directly affect the milk production of cows in the next parity and the incidence of postpartum metabolic diseases (Kok et al., 2017). Similarly, cow dry period body condition scores (BCS) impact on blood biochemistry, liver triacylglycerol, and muscular monocarboxylate transporter-1 mRNA expression (Triwutanon and Rukkwamsuk, 2021). Additionally, relationships between plasma total antioxidant capacity (TAC) of and physiological stages such as dry period, have been evaluated (Omidi et al., 2017).

At present, most comparative studies on the perinatal and dry periods of dairy cows have rarely been analyzed at the molecular level. The multi-omics technology that has emerged in the past 10 years provides us with ideas for analyzing the physiological changes of dairy cows from the molecular level. As an emerging research hotspot, circRNA has also become the object of our attention. CircRNAs varies greatly in different developmental stages and tissue expression abundance (Gruner et al., 2016), has high tissue specificity, and is highly conserved in different species (Cortés-López et al., 2018). The biological production of circRNA competes with linear splicing and can regulate the production of linear RNA in cis-genes. During the regulation process of the body, covalently closed circular structures are formed through reverse splicing mechanism in cells, which are selectively loaded into extracellular vesicles such as exosomes and enter various body fluids for intercellular communication, exerting regulatory effects on distant cells or tissues (Zhang et al., 2025).

The mammary gland is an important organ in Holstein cows as it is required for calf survival, passive immunity and early nutrition, and consumer products. The growth and development of the mammary glands are regulated by a variety of hormones (Rowson et al., 2012). And the expression changes of specific miRNAs also have certain impacts on the differentiation of mammary gland and epithelial cells (Nagaoka et al., 2013; Chen et al., 2020). In the study of circular RNA function in mammary gland tissue, researchers found that the expression level of specific circular RNAs (such as circR3HCC1) in the mammary glands of cows and goats is closely related to the synthesis of milk fat and milk protein. They directly affect the nutritional composition of milk by regulating downstream genes (Marei et al., 2025). Similarly, we compared circular RNAs in breast tissues from early lactation and non-lactating Holstein bovine and found that 87 circRNAs were significantly differentially expressed (Liang et al., 2022). Clearance of the above results, we speculate that there are also some special circRNAs in the perinatal and dry period mammary gland tissues of dairy cows. These special circRNAs have the potential to be molecular targets for us to study the different physiological characteristics of dairy cows during the perinatal and dry periods.

Therefore, this study used high-throughput RNA sequencing (RNA-seq) to study the expression profile of cirRNA from Holstein cows during perinatal and dry period, and identified the differential expressed cicrRNAs. In addition, gene ontology (GO) and Kyoto Encyclopedia of Genes and Genomes (KEGG) pathway enrichment analysis were performed for the parental mRNA genes of the differentially expressed cirRNA to investigate their potential roles. At the same time, we also predict the ceRNA mechanism between miRNA and circRNA. Through the characteristics of circRNAs in the perinatal and dry periods, circRNAs are expected to become novel molecular targets for the study of perinatal and dry periods diseases in dairy cows, and provide new research ideas for dairy cow genetic breeding.

## 2 Materials and methods

### 2.1 Statement of animal ethics

All experiments were conducted in accordance with the Guidelines for the Care and Use of Experimental Animals established by the Ministry of Science and Technology of the People’s Republic of China (Approval No. 2006–398). The collection of mammary gland tissue samples complied with the ethical standards governing experimental animal welfare. The experimental animal production license (SYDW-2019005) was obtained, and the study was approved by Yangzhou University, Yangzhou, China.

### 2.2 Tissue samples collection

Mammary gland tissue samples were collected from three Holstein cows during early lactation (n = 3; 7 days prepartum) and three during the non-lactating period (n = 3; 315 days postpartum) at a large-scale dairy farm in Jiangsu Province. Prior to tissue collection, milk was completely evacuated from the mammary glands of lactating cows confirmed to be free of mastitis. The biopsy procedure followed the methodology described by Li et al. (2016).

### 2.3 RNA preparation and circRNA sequencing

Mammary tissue samples (10–20 mg) were lysed in 300 μL RL buffer, homogenized, and incubated with 590 μL RNase-free ddH_2_O and 10 μL Proteinase K at 56 °C for 10–20 min. Total RNA was extracted using TRNzol reagent (Invitrogen, Carlsbad, CA) and purified with the RNAprep pure Tissue Kit (Tiangen, Beijing, China). The purified RNA was dissolved in DEPC-treated water, and its concentration (>400 ng/μL) and purity (A260/A280 = 1.9–2.2) were assessed using a NanoDrop® ND-1000 spectrophotometer (Thermo Scientific, DE).

Ribosomal RNA was removed from mRNA samples using a transcriptome isolation kit (Ribominus Bacteria 2.0, Thermo Fisher). The remaining RNA was used for library construction with the TruSeq RNA Library Preparation Kit (Illumina Inc., San Diego, US) and subjected to paired-end sequencing on an Illumina HiSeq Xten platform (Illumina Inc., San Diego, US) at Shanghai OE Biotechnology Company Ltd (Shanghai, China). Raw reads were filtered to remove low-quality reads (Q < 20, >50% of bases below threshold), reads with high error rate (>1%), ambiguous N bases, adaptor sequences, short reads (<20 bp), and rRNA-derived reads. Clean reads were used for circRNA identification using FIND_CIRC (Memczak et al., 2013), followed by prediction of known and novel circRNAs with CIRI software, referencing the circBase database (Gao et al., 2015). Chromosomal and length distributions of circRNAs were analyzed based on FIND_CIRC outputs. Junction read counts were normalized using DESeq, with expression levels estimated from mean base values. Differential expression was assessed using a negative binomial test (Anders and Huber, 2013), and differentially expressed circRNAs were identified based on fold change and significance thresholds.

### 2.4 Enrichment analysis

Kyoto Encyclopedia of Genes and Genomes (KEGG) and Gene Ontology (GO) enrichment analyses for the differential expressed circRNA source gene were performed using the DAVID biometric analysis tool (Kanehisa et al., 2008; Sun et al., 2014).

### 2.5 ceRNA

The miRNA-targets of each differentially expressed circRNA were predicted using the miRanda algorithm (Miranda et al., 2006) and the interaction network of the circRNAs and their target miRNAs was analyzed using starBase and then drawn using Cytoscape (Smoot et al., 2011). The calculation method of ceRNA_score and *P* value (Das et al., 2014) is as follows:

$$\text{ceRNA}\_\text{score}=\frac{\#\text{MRE}\_\text{for}\_\text{share}\_\text{miRNA}}{2a\#\text{MRE}\_\text{for}\_\text{circRNA}\_\text{miRNA}}$$

Where circRNA denotes the circRNA identifier; ceRNA_score refers to the predicted interaction score; #shared_miRNA indicates the number of shared miRNAs; miRNAs lists the names of those miRNAs; and pvalue represents the significance level for ceRNA prediction.

The calculation formula of *P*-value is as follows:

$$P=\sum_{i=m_{c}}^{\min\left(m_{p}.m_{n}\right)}\frac{\left(\begin{array}{c} m_{n}\\ i \end{array}\right)\left(\begin{array}{c} M_{t}-m_{n}\\ m_{p}-i \end{array}\right)}{\left(\begin{array}{c} M_{t}\\ m_{p} \end{array}\right)}$$

Where M_T_ represents the number of all miRNAs; m_p_ represents the number of miRNAs that regulate this mRNA. m_n_ represents the number of miRNAs that have regulatory effects on the circRNA; and m_c_ represents the number of common miRNAs.

## 3 Results

### 3.1 Identification and sequence characteristics of circRNAs in mammary tissue from Holstein cows

**FIGURE 1:**
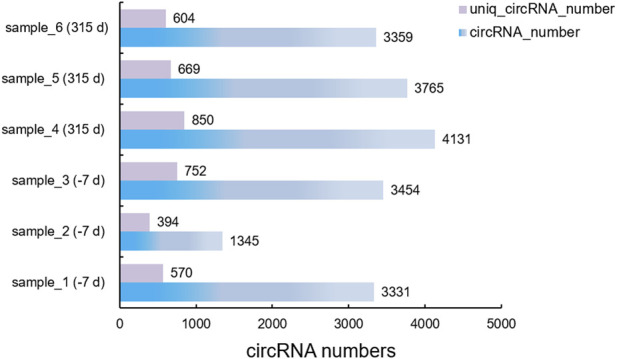
CircRNA numbers predicted in each sample. Legend: The horizontal axis is the number of circRNAs; the vertical axis shows the individual samples from cows on postpartum days −7 or 315; the numbers above each bar refer to the number of circRNAs predicted in each sample; the Uniq_circRNA_numbers refers to the number of circRNAs specifically predicted in each sample compared to other samples in the project.

**FIGURE 2:**
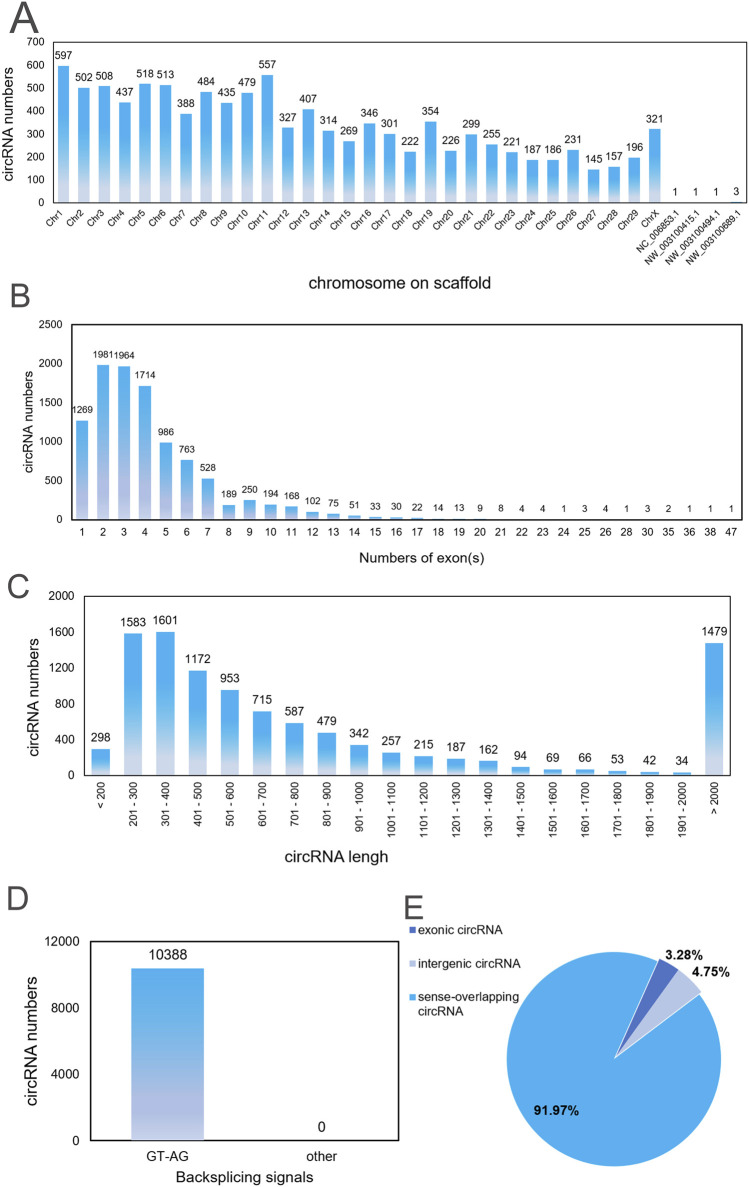
Identification, characterization, and chromosomal distribution of circRNAs. Legend: **(A)** Number of circRNAs per chromosome; **(B)** The number of exons by circRNAs; **(C)** The length distribution density of circRNAs; **(D)** Statistical diagram of circRNA shear signal, the number of circRNA on the vertical axis, and the type of shear signal on the horizontal axis; **(E)** Percentage of different types of circRNAs.

A total of 10,388 circRNAs were identified in mammary tissue RNA from Holstein cows by library construction, sequencing, and bioinformatics analysis. Count the number of circRNAs identified by each sample, as shown in Figure 1, the number of circRNAs predicted in the three samples at −7 days was 3331, 1345, 3454, and the number of unique circRNAs were 570, 394, 752, respectively. The number of circRNAs predicted in the three samples at 315 days was 4,131, 3765, 3359, and the number of unique circRNAs were 850, 669, 604, respectively. The chromosome 1 contained most circRNAs (n = 597) (Figures 2A). CircRNAs contains different number of exons, ranging from 1 to 47 and most of cirsRNAs harbored 1 to four exons (Figures 2B). The size of circRNAs ranged from 48 bp to 99,406 bp and the average size was 3027.79 bp. The circRNA lengths were mainly in the 201–500 bp, and lengths were more than 2000 bp (Figures 2C). Variable shear signals AT reverse shear sites in circRNA sequences were counted, and all of them were GT-AG (Figures 2D). There are three types of circRNA identified: exonic (3.28%), intergenic (4.75%), and sense-overlapping (91.97%) (Figures 2E).

### 3.2 Differential expression of circRNAs analysis in mammary tissue from Holstein cows

**FIGURE 3:**
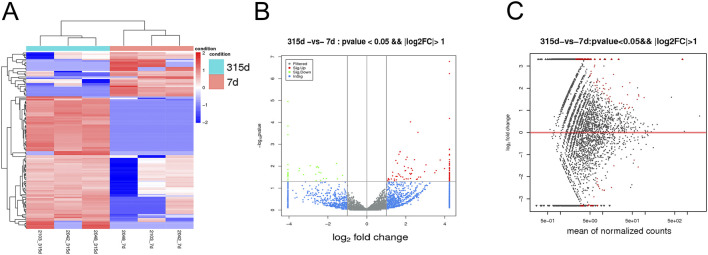
Differentially expressed circRNAs in mammary tissue from Holstein cows. Legend: **(A)** Differential circRNA expression level clustering. Red indicates high expression and blue indicates low expression. **(B)** Gray and blue circRNAs with non-significant differences, red and green circRNAs with upregulated and downregulated significant differences, respectively. The X-axis is the display of log2 Fold Change, and the Y-axis is the display of log_10_ *P* - value. **(C)** The X-axis is the mean expression of all samples used for comparison after standardization, and the Y-axis is Log2 Fold Change, and the red highlights are significant difference expressed circRNAs.

Compared with dry-period, 132 circRNAs with significantly different expressions were identified in perinatal-period, 99 of which were upregulated and 33 were downregulated (Supplementary Table S1). Unsupervised hierarchical clustering of differentially expressed circRNAs was carried out, the distance between pairs of multiple samples was calculated to form a distance matrix, and the expression of selected differential circRNAs was used to calculate the direct correlation of samples. The two clusters are up- and downregulated circRNAs, clustered in the same cluster may have similar biological functions (Figure 3).

### 3.3 circRNA enrichment

**FIGURE 4:**
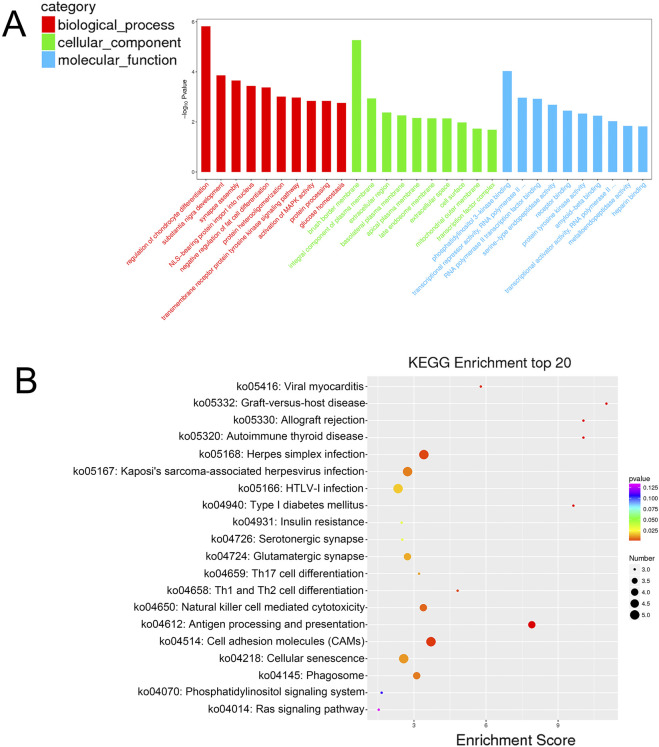
Go and KEGG Enrichment analysis. Legend: **(A)** The basic information for each node is displayed in the corresponding graph, which is the GO ID and GO term. The X-axis is the Go entry name, and the Y-axis is -log10 *P* - value. **(B)** Top 20 categories of KEGG pathway analysis, the X-axis Enrichment Score is the Enrichment Score. The larger the bubble is, the more circRNAs the item contains, and the color of the bubble changes from purple to blue to green to red. The smaller the Enrichment *P* - value is, the greater the significance is.

To investigate the possible functions of differentially expressed circRNAs, GO enrichment analysis was performed on the target genes of differentially expressed circRNAs. Through the enrichment analysis of 1094 GO term, revealed the enrichment of circRNAs in the proliferation and differentiation of cells, such as regulation of chondrocyte differentiation and integral component of plasma membrane, phosphatidylinositol 3-kinase binding (Figures 4A). The significantly enriched pathways further indicate that circRNAs play important roles in immunity and infection, such as cell adhesion molecules (CAMs), herpes simplex infection and kaposi’s sarcoma-associated herpesvirus infection (Figures 4B).

### 3.4 circRNA-miRNA interaction research

**FIGURE 5:**
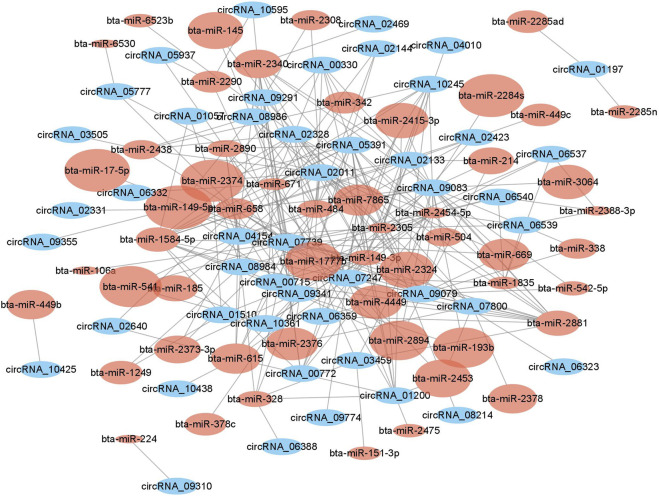
Top300 miRNA-circRNA ceRNA network.

Hypergeometric distribution testing was used to identify miRNAs with a large influence in differential circRNAs. The result of the calculation returns a *P*-value for enrichment significance. For the total difference circRNA enrichment results, the top 300 miRNA-circRNA interaction pairs with smaller *P* value were extracted by *P* value ordered, and the circRNA-miRNA target interaction network diagram was plotted using Cytoscape software (Figure 5).

## 4 Discussion

Cows are a significant source of dairy products and an ideal large animal model to study the transcriptome and expression characteristics of mammary tissues. Perinatal and dry period, as important physiological stages of cow recovery, play a key role in the prevention of immunity, lactation, and postpartum diseases. Therefore, it is necessary to explore the characteristics of the molecular mechanism of dairy cows at these stage. As a new type of endogenous non-coding RNA, circRNAs have been studied in humans (Yang et al., 2021), mice (Fan et al., 2015), pigs (Sun et al., 2017), cattle (Li et al., 2018), sheep (Wang et al., 2021), and other species (Zhang et al., 2019).

High-throughput sequencing was used to explore the presence and expression of circRNAs in mammary tissues from Holstein cows during perinatal and dry period, and to screen and identify circRNAs that may play an important role in lactation. Through systematic identification and analysis of circRNAs, it was found that 1716 and 2,123 unique circRNAs were predicted in mammary tissues from Holstein cows during perinatal and dry period. More interestingly, a total of 132 differential expressions were found in the two sets of circRNAs. We previously identified circRNAs in Holstein cows' mammary tissues during early lactating and non-lactating and found 10,686 circRNAs were predicted in the mammary tissue of Holstein cows at 30 days and 315 days postpartum (Liang et al., 2022). In sheep mammary gland tissues, Hao et al. (2020) identified 4,906 circRNAs in two sheep mammary gland tissues with different lactation performances and 33 of these were differentially expressed between breeds. Another study showed that 6,621 circRNAs were differentially expressed in the mammary tissue of Holstein cows at postpartum 90 days and 250 days, of which 2,231 were co-expressed (Zhang et al., 2016). Through these different results, we found that different physiological stages have a certain influence on the expression of circRNAs.

The first chromosome of cows contains the most circRNAs. This may be related to gene density and functional enrichment. Cow chromosome 1 has the most microsatellite loci and is one of the largest chromosomes in cattle, carrying the highest density of protein coding genes. Due to the fact that the vast majority of circular RNAs come from exons of protein coding genes, the denser the region of genes, the greater the potential for circular RNA production (Qi et al., 2013). Most circRNAs are short in length and are concentrated between 201 and 500 bp, while some circRNAs were greater than 2 kb. This is also consistent with many research findings (Liang et al., 2022). In addition, we found three different types of circRNAs in mammary tissue, of which 91.97% belonged to sense-overlapping, while Wang et al. (2021) identified six types of circRNAs in sheep mammary tissue, among which EciRNA was the main one. Sense-overlapping circRNAs (sometimes defined as EIciRNA) have overlapping regions with mRNA exons and are transcribed in the same direction. Related studies have shown that long flank introns are considered crucial to exon cyclization, and they contain ALU repeats (Jeck et al., 2013) and possibly help determine the production rate of circRNAs (Ashwal-Fluss et al., 2014). Finally, introns between the encircled exons are retained, which Li termed EIciRNAs (Li et al., 2015). Most of the circRNAs in this study are sense - overlapping, which may be related to the above reasons.

GO and KEGG enrichment analysis can illustrate the related functions of genes. Enrichment analysis revealed the enrichment of circRNAs in proliferation and differentiation of cells, such as regulation of chondrocyte differentiation and integral component of plasma membrane, phosphatidylinositol 3-kinase binding. The significantly enriched pathways further indicate that circRNAs play important roles in immunity and infection, such as cell adhesion molecules (CAMs), herpes simplex infection and Kaposi’s sarcoma-associated herpesvirus infection. Cows suffer severe physiological stress during the perinatal period, during which increased nutrient requirements, loss of appetite and milk production lead to energy deficiency in cows, which leads to negative energy balance and metabolic disorders (Lv et al., 2022). Compared with the dry milk period, perinatal cows need to be prepared for lactation initiation, and mammary gland related functions are more active, which may be one of the reasons why GO enrichment is associated with cell proliferation and differentiation. In addition, the immunity of dairy cows in the perinatal period began to decline, and immune-related neutrophils, lymphocytes, and monocytes were suppressed in the perinatal period (Khan et al., 2020). The KEGG pathways of immune response is the most significantly pathways, which is important for fighting pathogen infections (Korkmaz et al., 2018), and the special physiological characteristics of the perinatal period led to enrichment in pathways related to inflammation and immunity.

Studies have shown that circRNA can act as a “sponge” competitive binding target site for miRNA, affecting the translation of mRNA, representing a new class of ceRNA regulators (Zhao et al., 2020). Sun et al. (2017) identified 68 sponge modulators participating in 26 miRNA-mediated ceRNA interactions, including 40 circRNAs, and 9 mRNAs. Li et al. (2018) found that circFGFR4 binding miR-107 promotes cell differentiation via targeting Wnt3a in bovine primary myoblasts. Another study also reported circ11103 regulates milk fat metabolism in dairy cows through the ceRNA mechanism (Chen et al., 2021). Through the above studies, we found that in different organisms and different physio-logical stages, circRNA has a specific expression regulation mechanism and plays rich and important functions, not just as a by-product of transcription. We also proved this in the circRNA-miRNA interaction research. For the total difference circRNA enrichment results, the top 300 miRNA-circRNA interaction pairs with smaller *P* value were extracted by P value ordered, and the circRNA-miRNA target interaction network diagram was plotted using Cytoscape software. In the following study, based on the feature analysis of circRNAs and the enrichment of differentially expressed circRNAs, we will screen out specific differentially expressed circRNAs, miRNAs, and mRNAs with targeted relationships for functional validation and targeted relationship validation, further exploring how differentially expressed circRNAs affect milk production levels and quality in cows at different lactation stages.

## 5 Conclusion

In this study, through high-throughput sequencing of circRNAs in Holstein cow mammary tissues at perinatal and dry period, 10,388 circRNAs were detected, mainly distributed on chromosomes 1 and mainly sense-overlapping. Among the 132 differentially expressed circRNAs detected, enrichment analysis revealed the enrichment of circRNAs in proliferation and differentiation of cells and significantly enriched pathways further indicate that circRNAs play important roles in immunity and infection.

This study revealed the characteristics of DEcircRNAs in the mammary gland tissues of cows during the perinatal and dry milk periods. These DEcircRNAs can serve as molecular signals for the switching between the “stop milk production” and “prepare for lactation” states in the mammary gland of cows, directly participating in immune activation, inflammation regulation, and tissue remodeling processes. This provides a scientific basis for the development of new disease resistant breeding markers, early disease diagnosis tools, and precise health management strategies. The ultimate goal is to help cows navigate the high-risk stage of the perinatal period more smoothly, reduce disease occurrence, and improve animal welfare and production efficiency.

## Data Availability

The original contributions presented in the study are publicly available. This data can be found in the NCBI repository with the BioProject accession number PRJNA766152 and the BioSample accession number SAMN21619997.

## References

[B1] Anders S. Huber W. (2013). Differential expression of RNA-Seq data at the gene level–the DESeq package. Heidelberg, Germany. Eur. Mol. Biol. Lab. Embl. 02, 1–23.

[B2] Ashwal-Fluss R. Meyer M. Pamudurti N. R. Ivanov A. Bartok O. Hanan M. (2014). circRNA biogenesis competes with Pre-mRNA splicing. Mol. Cell 56, 55–66. 10.1016/j.molcel.2014.08.019 25242144

[B3] Chen Z. Chu S. Liang Y. Xu T. Sun Y. Li M. (2020). miR-497 regulates fatty acid synthesis *via* LATS2 in bovine mammary epithelial cells. Food and Funct. 11, 8625–8636. 10.1039/d0fo00952k 32935676

[B4] Chen Z. Lu Q. Liang Y. Cui X. Wang X. Mao Y. (2021). Circ11103 interacts with miR-128/PPARGC1A to regulate milk fat metabolism in dairy cows. J. Agric. Food Chem. 69 (15), 4490–4500. 10.1021/acs.jafc.0c07018 33826315

[B5] Cortés-López M. Gruner M. R. Cooper D. A. Gruner H. N. Voda A. I. van der Linden A. M. (2018). Global accumulation of circRNAs during aging in Caenorhabditis elegans. BMC Genomics 19 (1), 1–12. 10.1186/s12864-017-4386-y 29298683 PMC5753478

[B6] Das S. Ghosal S. Sen R. Chakrabarti J. (2014). lnCeDB: database of human long noncoding RNA acting as competing endogenous RNA. PLoS One 9 (6), 98965. 10.1371/journal.pone.0098965 24926662 PMC4057149

[B7] Drackley J. K. (1999). Biology of dairy cows during the transition period: the final frontier? J. Dairy Sci. 82 (11), 2259–2273. 10.3168/jds.S0022-0302(99)75474-3 10575597

[B8] Fan X. Zhang X. Wu X. Guo H. Hu Y. Tang F. (2015). Single-cell RNA-seq transcriptome analysis of linear and circular RNAs in mouse preimplantation embryos. Genome Biol. 16 (1), 148. 10.1186/s13059-015-0706-1 26201400 PMC4511241

[B9] Gao Y. Wang J. F. Zhao F. Q. (2015). CIRI: an efficient and unbiased algorithm for *de novo* circular RNA identification. Genome Biol. 16 (1), 4–19. 10.1186/s13059-014-0571-3 25583365 PMC4316645

[B10] Gruner H. Cortés-López M. Cooper D. A. Bauer M. Miura P. (2016). CircRNA accumulation in the aging mouse brain. Sci. Reports 6 (1), 38907. 10.1038/srep38907 27958329 PMC5153657

[B11] Hao Z. Zhou H. Hickford J. G. H. Gong H. Wang J. Hu J. (2020). Identification and characterization of circular RNA in lactating mammary glands from two breeds of sheep with different milk production profiles using RNA-Seq. Genomics 112 (3), 2186–2193. 10.1016/j.ygeno.2019.12.014 31866420

[B12] Jeck W. R. Sorrentino J. A. Wang K. Slevin M. K. Burd C. E. Liu J. (2013). Circular RNAs are abundant, conserved, and associated with ALU repeats. RNA 19, 141–157. 10.1261/rna.035667.112 23249747 PMC3543092

[B13] Jiang L. Y. Sun H. Z. Guan R. W. Shi F. Zhao F. Q. Liu J. X. (2022). Formation of blood neutrophil extracellular traps increases the mastitis risk of dairy cows during the transition period. Front. Immunol. 13, 880578. 10.3389/fimmu.2022.880578 35572521 PMC9092530

[B14] Kanehisa M. Araki M. Goto S. Hattori M. Hirakawa M. Itoh M. (2008). KEGG for linking genomes to life and the environment. Nucleic Acids Research 36 (Suppl. 1), 480–484. 10.1093/nar/gkm882 18077471 PMC2238879

[B15] Khan M. Z. Zhang Z. Liu L. Wang D. Mi S. Liu X. (2020). Folic acid supplementation regulates key immunity-associated genes and pathways during the periparturient period in dairy cows. Asian-Australasian J. Animal Sci. 33 (9), 1507–1519. 10.5713/ajas.18.0852 31010964 PMC7468170

[B16] Kok A. van Hoeij R. J. Tolkamp B. J. Haskell M. J. Knegsel A. T. M. van de Boer I. J. M. (2017). Behavioural adaptation to a short or no dry period with associated management in dairy cows. Appl. Animal Behav. Sci. 186, 7–15. 10.1016/j.applanim.2016.10.017

[B17] Korkmaz F. T. Elsasser T. H. Kerr D. E. (2018). Variation in fibroblast expression of toll-like receptor 4 and lipopolysaccharide-induced cytokine production between animals predicts control of bacterial growth but not severity of Escherichia coli mastitis. J. Dairy Sci. 101 (11), 10098–10115. 10.3168/jds.2017-14372 30172411

[B18] LeBlanc S. J. Lissemore K. D. Kelton D. F. Duffield T. F. Leslie K. E. (2006). Major advances in disease prevention in dairy cattle. J. Dairy Sci. 89 (4), 1267–1279. 10.3168/jds.S0022-0302(06)72195-6 16537959

[B19] Li Z. Huang C. Bao C. Chen L. Lin M. Wang X. (2015). Exon-intron circular RNAs regulate transcription in the nucleus. Nat. Struct. and Mol. Biol. 22, 256–264. 10.1038/nsmb.2959 25664725

[B20] Li C. Cai W. Zhou C. Yin H. Zhang Z. Loor J. J. (2016). RNA-Seq reveals 10 novel promising candidate genes affecting milk protein concentration in the Chinese Holstein population. Sci. Rep. 6, 26813. 10.1038/srep26813 27254118 PMC4890585

[B21] Li H. Wei X. Yang J. Dong D. Hao D. Huang Y. (2018). CircFGFR4 promotes differentiation of myoblasts *via* binding miR-107 to relieve its inhibition of Wnt3a. Mol. Therapy-Nucleic Acids 11, 272–283. 10.1016/j.omtn.2018.02.012 29858062 PMC5992882

[B22] Liang Y. Gao Q. Wang H. Guo M. Arbab A. A. I. Nazar M. (2022). Identification and characterization of circular RNAs in mammary tissue from holstein cows at early lactation and non-lactation. Biomolecules 12 (3), 478. 10.3390/biom12030478 35327670 PMC8946036

[B23] Lv D. Gao J. Wu Z. Sun Z. Hao L. Liu S. (2022). Multiomic analyses reveal the effects of supplementing phytosterols on the metabolic function of the rumen microbiota in perinatal cows. Appl. Environ. Microbiol. 88 (15), e0099222. 10.1128/aem.00992-22 35856688 PMC9361816

[B24] Marei S. Maatouk N. AbouHaidar M. Talhouk R. (2025). Developmental regulation of circRNAs in normal and diseased mammary gland: a focus on circRNA-miRNA networks. J. Mammary Gl. Biol. Neoplasia 30 (1), 8. 10.1007/s10911-025-09580-w 40314719 PMC12048424

[B25] Memczak S. Jens M. Elefsinioti A. Torti F. Krueger J. Rybak A. (2013). Circular RNAs are a large class of animal RNAs with regulatory potency. Nature 495, 333–338. 10.1038/nature11928 23446348

[B26] Miranda K. C. Huynh T. Tay Y. Ang Y. S. Tam W. L. Thomson A. M. (2006). A pattern-based method for the identification of MicroRNA binding sites and their corresponding heteroduplexes. Cell 126 (6), 1203–1217. 10.1016/j.cell.2006.07.031 16990141

[B27] Mulligan F. J. Doherty M. L. (2008). Production diseases of the transition cow. Veterinary J. 176 (1), 3–9. 10.1016/j.tvjl.2007.12.018 18342556

[B28] Nagaoka K. Zhang H. Watanabe G. Taya K. (2013). Epithelial cell differentiation regulated by MicroRNA-200a in mammary glands. PLoS One 8 (6), e65127. 10.1371/journal.pone.0065127 23750238 PMC3672172

[B29] Omidi A. Fathi M. H. Parker M. O. (2017). Alterations of antioxidant status markers in dairy cows during lactation and in the dry period. J. Dairy Res. 84 (1), 49–53. 10.1017/S0022029916000753 28007040

[B30] Qi W. H. Jiang X. M. Xiao G. S. Huang X. Y. Du L. M. (2013). Seeking and bioinformatics analysis of microsatellite sequence in the genomes of cow and sheep. Acta Veterinaria Zootechnica Sinica 44 (11), 1724–1733.

[B31] Rowson A. R. Daniels K. M. Ellis S. E. Hovey R. C. (2012). Growth and development of the mammary glands of livestock: a veritable barnyard of opportunities. Seminars Cell and Dev. Biol. 23 (5), 557–566. 10.1016/j.semcdb.2012.03.018 22504021

[B32] Smoot M. E. Ono K. Ruscheinski J. Wang P. L. Ideker T. (2011). Cytoscape 2.8: new features for data integration and network visualization. Bioinformatics 27 (3), 431–432. 10.1093/bioinformatics/btq675 21149340 PMC3031041

[B33] Sun J. Wang S. Li C. Ren Y. Wang J. (2014). Novel expression profiles of microRNAs suggest that specific miRNAs regulate, gene expression for the sexual maturation of female Schistosoma japonicum after pairing. Parasites and Vectors 7 (1), 177. 10.1186/1756-3305-7-177 24721600 PMC4021575

[B34] Sun J. Xie M. Huang Z. Li H. Chen T. Sun R. (2017). Integrated analysis of non-coding RNA and mRNA expression profiles of 2 pig breeds differing in muscle traits. J. Animal Sci. 95 (3), 1092–1103. 10.2527/jas.2016.0867 28380516

[B35] Trevisi E. Amadori M. Cogrossi S. Razzuoli E. Bertoni G. (2012). Metabolic stress and inflammatory response in high-yielding, periparturient dairy cows. Res. Veterinary Sci. 93 (2), 695–704. 10.1016/j.rvsc.2011.11.008 22197526

[B36] Triwutanon S. Rukkwamsuk T. (2021). Effects of body condition at far-off dry period on blood biochemistry, liver triacylglycerol and muscular monocarboxylate transporter-1 mRNA expression in tropical Holstein dairy cows during peripartum period. Animal Sci. J. 92 (1), e13671. 10.1111/asj.13671 34931748

[B37] Wang J. Zhou H. Hickford J. G. H. Hao Z. Gong H. Hu J. (2021). Identification and characterization of circular RNAs in mammary gland tissue from sheep at peak lactation and during the nonlactating period. J. Dairy Sci. 104 (2), 2396–2409. 10.3168/jds.2020-18911 33246614

[B38] Yang X. Ye T. Liu H. Lv P. Duan C. Wu X. (2021). Expression profiles, biological functions and clinical significance of circRNAs in bladder cancer. Mol. Cancer 20 (1), 4–29. 10.1186/s12943-020-01300-8 33397425 PMC7780637

[B39] Zhang C. Wu H. Wang Y. Zhu S. Liu J. Fang X. (2016). Circular RNA of cattle casein genes are highly expressed in bovine mammary gland. J. Dairy Sci. 99 (6), 4750–4760. 10.3168/jds.2015-10381 27040791

[B40] Zhang Y. Wang L. Qiu L. Pan R. Bai H. Jiang Y. (2019). Expression patterns of novel circular RNAs in chicken cells after avian leukosis virus subgroup J infection. Gene 701, 72–81. 10.1016/j.gene.2019.03.030 30898701

[B41] Zhang N. Wang X. J. Li Y. Lu Y. W. Sheng C. C. Sun Y. M. (2025). Mechanisms and therapeutic implications of gene expression regulation by circRNA-protein interactions in cancer. Commun. Biology 8, 77. 10.1038/s42003-024-07383-z 39825074 PMC11748638

[B42] Zhao J. Mu L. Wang Z. Fang X. He X. Zhang X. (2020). The potential roles of circular RNAs in osteonecrosis of the femoral head. Mol. Medicine Reports 21 (2), 533–539. 10.3892/mmr.2019.10866 31974613 PMC6947852

